# CRISPR/Cas9-Mediated TERT Disruption in Cancer Cells

**DOI:** 10.3390/ijms21020653

**Published:** 2020-01-19

**Authors:** Luan Wen, Changzhi Zhao, Jun Song, Linyuan Ma, Jinxue Ruan, Xiaofeng Xia, Y. Eugene Chen, Jifeng Zhang, Peter X. Ma, Jie Xu

**Affiliations:** 1 Center for Advanced Models and Translational Sciences and Therapeutics, University of Michigan, Ann Arbor, MI 48109, USA; changzhi@med.umich.edu (C.Z.); songjun@med.umich.edu (J.S.); linyuanm@med.umich.edu (L.M.); ruanjinxue2008@126.com (J.R.); echenum@med.umich.edu (Y.E.C.); jifengz@med.umich.edu (J.Z.); 2 Research & Development, ATGC Inc. 100 E Lancaster Avenue, LIMR Building Lab129, Wynnewood, PA 19096, USA; xxia@atgcinc.com; 3 Department of Biologic and Materials Sciences, University of Michigan, Ann Arbor, MI 48109, USA; mapx@umich.edu

**Keywords:** CRISPR/Cas9, gene editing, telomerase, TERT, cancer therapy

## Abstract

Mammalian telomere lengths are primarily regulated by telomerase, a ribonucleoprotein consisting of a reverse transcriptase (TERT) and an RNA subunit (TERC). TERC is constitutively expressed in all cells, whereas TERT expression is temporally and spatially regulated, such that in most adult somatic cells, TERT is inactivated and telomerase activity is undetectable. Most tumor cells activate TERT as a mechanism for preventing progressive telomere attrition to achieve proliferative immortality. Therefore, inactivating TERT has been considered to be a promising means of cancer therapy. Here we applied the CRISPR/Cas9 gene editing system to target the TERT gene in cancer cells. We report that disruption of TERT severely compromises cancer cell survival in vitro and in vivo. Haploinsufficiency of TERT in tumor cells is sufficient to result in telomere attrition and growth retardation in vitro. In vivo, TERT haploinsufficient tumor cells failed to form xenograft after transplantation to nude mice. Our work demonstrates that gene editing-mediated TERT knockout is a potential therapeutic option for treating cancer.

## 1. Introduction

Telomeres are the unique structures in eukaryotic cells that maintain chromosome integrity. Vertebrates telomeres consist of tandem repeats of 5′-TTAGGG-3′ sequences. Shelterin proteins including TRF1, TRF2, POT1, TIN2, TPP1, and RAP1 bind telomeres to protect chromosome ends [1]. In most somatic cells where telomere maintenance mechanisms (TMM) are silent, telomeres are shortened at each cellular division due to the end replication problem, progressively leading to replicative senescence [2]. In stem cells and cancer cells, TMM is activated to overcome telomeric DNA attrition and replicative senescence [3]. There are two major TMM pathways: the primary one is telomerase-dependent, and the other is the telomerase-independent alternative lengthening of telomeres (ALT). 

Telomerase is a ribonucleoprotein consisting of an reverse transcriptase subunit encoded by TERT [4] and an RNA subunit encoded by TERC [5]. Telomerase possesses the ability to elongate telomeres by adding telomeric repeats to the end of the 3′ G-rich strand of eukaryotic chromosomes. While TERC is constitutively expressed, TERT is suppressed in most somatic cells [6]. Most cancer cells (80–90%) activate TERT and subsequently telomerase to gain immortalization [7,8,9]. High telomerase activity is considered one of the six hallmarks of cancer proposed by Hanahan and Weinberg in 2000 [10]; therefore, targeting TERT/telomerase has long been considered to be a promising target for cancer drug development. To date, most drug development activities have focused on small molecule compounds [11,12] and oligonucleotides [13,14] as potential telomerase inhibitor drugs. 

CRISPR (clustered regularly interspaced short palindromic repeats)/Cas9 (CRISPR Associated Protein 9) has become a mainstream tool in biomedical research in recent years [15] and holds potential for gene editing-based therapeutics [16,17]. Cas9 is also a ribonucleoprotein consisting of a nuclease component and a guide RNA (gRNA). After gRNA binds to the target sequence, the Cas9 nuclease generates a double stranded breaks (DSB). These DSBs can be repaired by the error-prone non-homologous end joining (NHEJ) pathway or the homology directed repair (HDR) pathway, which have been exploited to introduce knockout (KO) or precise knock-in (KI) mutations, respectively [18]. 

We previously reported efficient Cas9-mediated gene KO and KI in creating animal and cellular models [19,20,21]. In the present work, we hypothesize that Cas9-mediated gene KO of TERT will compromise cancer cell survivability. While several groups have utilized Cas9 to gene edit the promoter sequences of TERT [22,23,24,25], only one report mentioned TERT gene KO however the detailed strategy and efficiency were not described [26]. Here we report an efficient Cas9 editing strategy on the TERT gene and its efficacy in suppressing tumor cell growth in vitro and in vivo. 

## 2. Results

### 2.1. Evaluation of Single gRNA Strategies to Target the TERT Exon in Cancer Cells

To ensure that TERT is a valid target, we first used quantitative RT-PCR to determine the expression levels of TERT in these cells. As expected, TERC is constitutively expressed in all cells examined; whereas TERT is expressed in cancer cells (Hela, PANC1 and SUM15) and human induced pluripotent stem cells (iPSCs), but not in primary cells such as human aortic smooth muscle cells (HASMCs) (Supplementary Figure S1A). The relative telomere length/content normalized to single copy gene, referred to as the T/S ratio [27], measured by quantitative PCR is highest in the iPSCs, moderate in HASMCs, but extremely low in all cancer cells (Supplementary Figure S1B). These findings are consistent with prior reports that cancer cells carry short telomeres and that TERT is activated in cancer cells as a means to achieve immortality [28], and support our strategy to disrupt TERT for suppressing cancer cells. 

**Figure 1:**
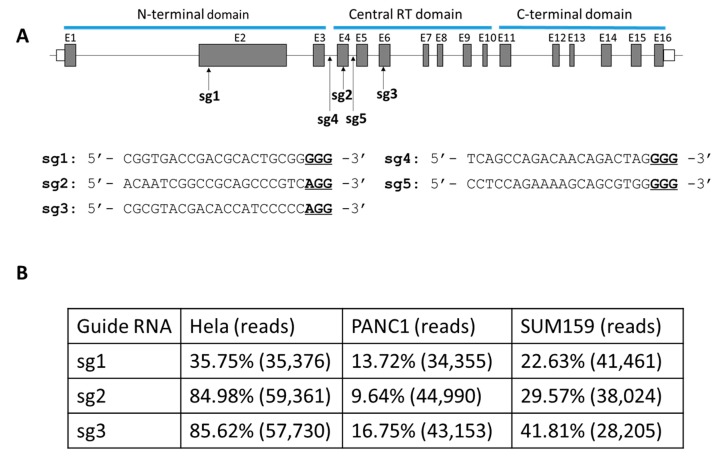
Design and efficiencies of Cas9 gRNAs targeting TERT. (**A**) Top: illustration of gRNAs (sg1 to sg5) targeting TERT. E#: Exon#. Bottom: gRNA sequences. Underlined: PAM. (**B**) Indel efficiencies of sg1, sg2 and sg3 determined by deepseq in Hela, PANC1 and SUM159 cells. Total deepseq reads for each locus were shown in parentheses.

The dominant Cas9-mediated gene disruption strategy utilizes one gRNA to target an exon of the gene. Here we designed three gRNAs (sg1, sg2 and sg3) targeting the human TERT gene exon 2 (E2), exon4 (E4) and exon 6 (E6), respectively (Figure 1A). Because our hypothesis is that TERT gene disruption may offer a universal approach to treat cancer, we selected three different cancer cell lines to evaluate the efficiencies of these gRNAs: a cervical cancer cell line Hela, a pancreatic cancer cell line PANC1, and a breast cancer cell line SUM159. 

The targeting efficiencies of each gRNA, as determined by the insertion and/or deletion (indel) rates at the target locus, were evaluated by deep sequencing (deepseq). Sg1, sg2 and sg3 all worked well in Hela cells, with indel rates of 35.75%, 84.98% and 85.62%, respectively (Figure 1B). The same gRNAs (sg1, sg2 and sg3) led to lower indel rates in SUM159 cells (22.63 to 41.81%) and even lower in PANC1 cells (9.64 to 16.75%), indicating that the gRNA indel efficiencies may be cell line dependent. 

### 2.2. Development of an Exon Removal Strategy to Knockout TERT

We next developed an exon removal strategy to delete an entire exon to ensure functional gene knockout. To achieve this, two gRNAs (sg4 and sg5) targeting the flanking introns of TERT exon 4 (E4) were used (Figure 1A). Single cell-derived colonies (*n* = 21 for Hela cells, *n* = 17 for SUM159 cells and *n* = 24 for PANC1 cells) were established after gene editing. Genomic DNA harvested from individual clones were used for genotyping to evaluate the exon removal efficiency. Interestingly, and unexpectedly, while TERT^+/−^ (referred to as “TERT haploinsufficiency” interchangeably hereafter) and TERT^+/+^ clones were obtained, we were unable to establish any TERT homozygous knockout (TERT^−/−^) clones from any of these three types of cancer cells, indicating that TERT^−/−^ tumor cells have extremely low survival rates in vitro. These TERT^+/+^, i.e., wild-type (WT), clones derived post editing (WTPE) were kept and used as WT controls in follow-up experiments. 

**Figure 2:**
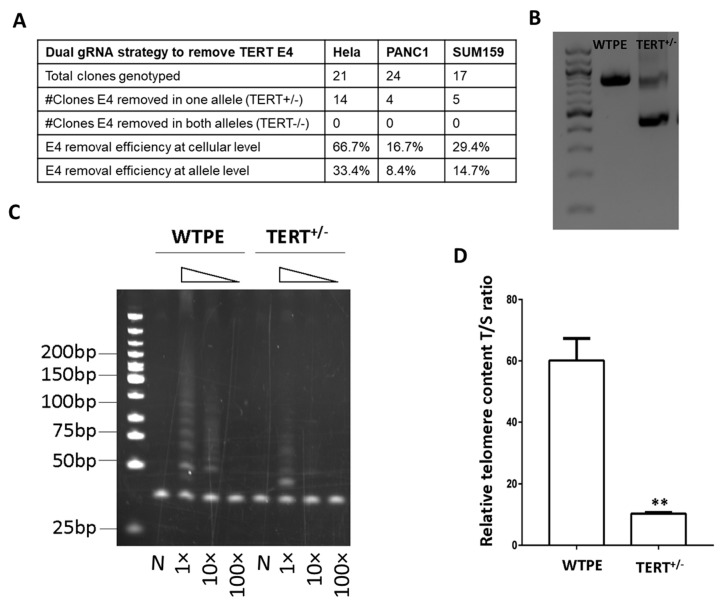
Generation of TERT^+/−^ tumor cells by the exon removal strategy using sg4 and sg5. (**A**) Efficiencies of E4 removal by using both sg4 and sg5. (**B**) Representative genotyping results of a TERT^+/−^ Hela cell clone. M: molecule weight markers. (**C**) Telomerase activity in WT and TERT^+/−^ Hela cells at 1×, 10× and 100× dilutions determined by the TRAP assay. N: heat inactivated negative control. M: molecule weight markers. (**D**) Relative telomere content T/S ratio in WT and TERT^+/−^ Hela cells. ** *p* < 0.01.

The exon removal efficiencies (Figure 2A,B) were highest in the Hela cells (66.7% at cellular level or 33.4% at allele level), lower in SUM159 cells (29.4% at cellular level or 14.7% at allele level) and lowest in PANC1 cells (16.7% at cellular level or 8.4% at allele level). Among the three cancer lines that we tested, Hela cells appeared to be the most amenable one for gene editing, and were selected for subsequent experiments. 

One concern for Cas9-based therapy is the off-target editing. We evaluated top potential off-target mutations for sg4 (*n* = 9) and sg5 (*n* = 9) in Hela cells (Supplementary Table S1). No off-target mutations were detected. Although this result indicates that Cas9 mediated editing by using sg4 or sg5 comes with low off-target risks in the present work, we agree that whole genome sequencing is needed to evaluate their genotoxicity for any clinical applications [29]. 

These results show that the Cas9-based exon removal strategy can be used to effectively create TERT^+/−^ mutations in cancer cells.

### 2.3. Cas9-Mediated TERT Haploinsufficiency in Cancer Cells Leads to Lower Telomerase Activity and Shorter Telomeres

We proceeded with TERT^+/−^ and WTPE Hela cells to determine how TERT haploinsufficiency affects the telomerase activity and telomere lengths in these cells. Passage 2 cells were used, approximately 20 days post transfection/single cell clone derivation. Western blot assay show that the TERT protein expression was lowered in TERT^+/−^ Hela cells compared to the WTPE counterparts (Supplementary Figure S2), although the signals were not as strong as those observed in TERT^+/−^ vs. WTPE PANC1 cells, indicating a cell line difference in TERT expression levels. Nevertheless, the telomerase activity, as determined by the Telomerase Repeated Amplification Protocol (TRAP) assay [30], was lowered in the TERT^+/−^ Hela cells compared to that in WTPE cells (Figure 2C). Consistently, the T/S ratio, an indicator of the relative telomere length, is much lower in the TERT^+/−^ Hela cells than that in the WTPE cells (Figure 2D). 

These results show that TERT haploinsufficient is sufficient to result in lowered telomerase activity and shortened telomere lengths in tumor cells. 

### 2.4. Cas9-Mediated TERT Haploinsufficiency in Cancer Cells Leads to Retarded Growth and Enhanced Cell Death In Vitro

**Figure 3:**
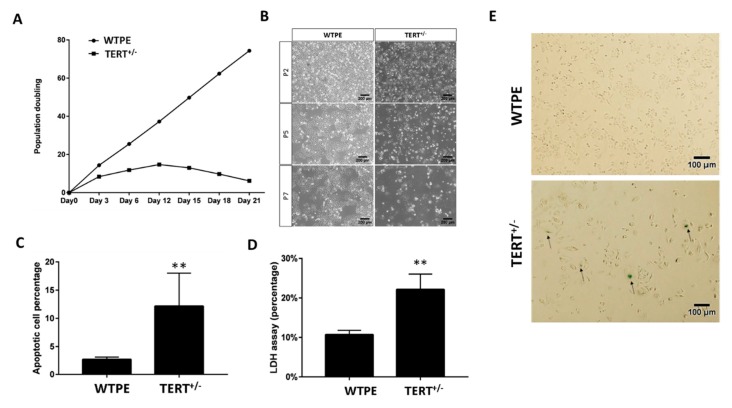
Retarded growth and increased cell death in Tert^+/−^ cancer cells. (**A**) Population doubling time of WT and TERT^+/−^ Hela cells. (**B**) Light microscopy images of WT and TERT^+/−^ Hela cells at Passage 2 (P2), P5 and P7. (**C**) β-gal staining of WT and TERT^+/−^ Hela cells. Arrows: example of severely senescent cells. (**D**) Cell death rates of WT and TERT^+/−^ Hela cells determined by LDH assay. (**E**) Quantification of flow cytometry analysis of annexin-V and propidium iodide (PI) staining of apoptotic cells in WT and TERT^+/−^ Hela cells. ** *p* < 0.01.

The cell proliferation, as measured by the population doubling time, was much slower in TERT^+/−^ than that in WTPE Hela cells in culture (Figure 3A). Consistently, TERT^+/−^ Hela cells were of lower density in culture than that of WTPE cells (Figure 3B,C). The size of TERT^+/−^ cells appeared to be much larger than that of WTPE cells, accompanied by stronger β-gal staining signals (Figure 3C), indicating a more severe extent of cellular senescent in TERT^+/−^ than that in the WTPE Hela cells [31]. Moreover, Pierce LDH Cytotoxicity Assay revealed higher cellular death rate in the TERT^+/−^ than that in the WTPE Hela cells (Figure 3D). Cellular apoptosis, evidenced by Annxin V and PI staining, is also enhanced in TERT^+/−^ than that in the WTPE Hela cells (Figure 3E). 

These data indicate that haploinsufficient TERT mutation causes retarded growth and eventually death in cancer cell cultures due to enhanced senescence and apoptosis.

### 2.5. Cas9-Mediated TERT Haploinsufficiency Severely Compromises the In Vivo Growth of Cancer Cell Xenograft

**Figure 4:**
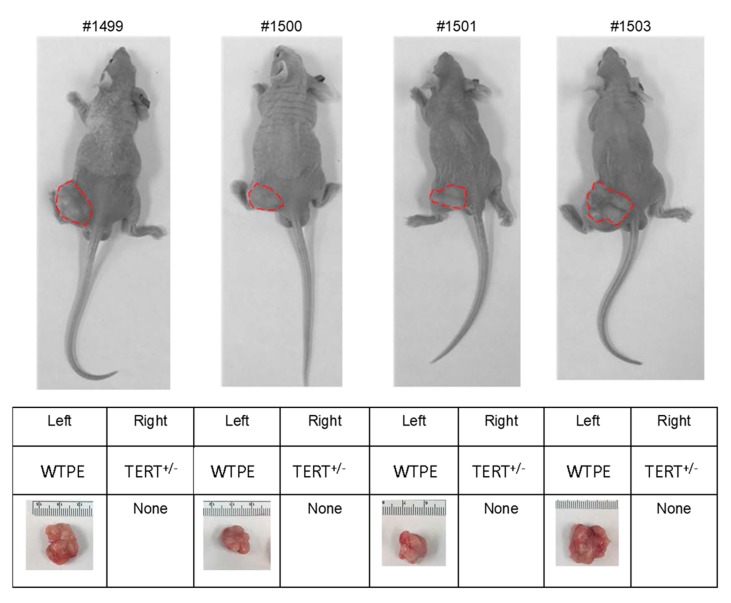
Xenotransplant of WT and TERT^+/−^ Hela cells in nude mice. Top: nude mice received TERT^+/−^ Hela cells on their right hind legs, and WT Hela cells on their left hind legs. Red circle: visible xenotransplant mass. Bottom: Summary table of xenotransplant results.

We next tested if TERT haploinsufficiency has effects on tumor cell survival in vivo using a tumor xenotransplant model in nude mice (*n* = 4). Each animal received ~1 × 10^6^ TERT^+/−^ Hela cells on its right hind leg and ~1 × 10^6^ WTPE Hela cells as control on its left hind leg. Six weeks post inoculation, we examined the sizes of the xenografts. As expected, the WTPE Hela cells grew into tumor mass (diameters ~2 cm) in all animals; whereas strikingly, the TERT^+/−^ Hela cells failed to form any tumor xenotransplant in any of these animals (Figure 4). 

This result supports our hypothesis that Cas9-mediated TERT haploinsufficiency can effectively suppress tumor cell growth in vivo. 

## 3. Discussion

Cancer is essentially a genetic disease caused by oncogenic mutations that transform normal cells to malignant cancer cells [32]. It has been demonstrated that Cas9 can be utilized to induce oncogenic mutations that lead to spontaneous cancer development in mouse models [33,34,35]. It is conceivable that gene editing tools can also be used to target cancer-related genes to treat or even cure cancer [35]. Yet there are several major technical challenges that remain to be addressed before this approach can be tested in clinical settings, including low precise gene editing efficiency, substantial off-target editing events, and perhaps most significantly the lack of an effective and safe delivery system to send gene editing nuclease elements to tumor cells [36]. While tremendous efforts have been dedicated towards solutions for these challenges, the present work worked on two prerequisites for gene editing-based cancer therapies: (i) identification and validation of tumor specific target gene(s); and (ii) development of efficient editing strategies on the target gene(s). 

Here we identified the TERT gene as the first choice to develop efficient gene editing strategies towards the goal of gene editing therapy for cancer. This is because tumor cells, up to 90% in all cancer types, activate TERT/telomerase to achieve immortality. As such, inhibiting TERT may provide a universal therapy for treating a wide spectrum of cancers [37]. 

Through the present work, we developed Cas9-based strategies to efficiently edit the TERT gene. Several prior reports have utilized Cas9 to target the promoter sequences of TERT to achieve ablation or enhancement of the TERT expression [22,23,24,25], but little information is available on targeting the TERT gene exons or introns. Here we report several efficient gRNAs targeting the TERT gene exons or intros with indel rates up to 85%. Beyond the conventional single gRNA mediated gene knockout approach, we worked to develop a dual gRNA gene knockout strategy. This is because while the single gRNA approach can be highly efficient, there are two potential risks. First, there is a one third chance that the resultant indels are in-frame, and these in-frame changes may not necessarily lead to gene knockout. Secondly, and more importantly, the indels on the exon will introduce new mutations, raising concerns especially when this gene is cancer associated. One way to circumvent these risks is to use a dual sgRNA strategy. In our work, two gRNAs were used, each targeting an intron region flanking the TERT E4. The action of both gRNAs would remove E4, ensuring the functional gene knockout. In the event that the large fragment (e.g., E4) deletion does not take place, no changes in the coding sequence are expected because the indels caused by each single gRNA would be on the introns, thereby avoiding secondary undesirable mutations on the exons, a desirable feature in clinical applications. The dual gRNA approach for removing TERT E4 presented here may prove to be an effective and safe method for gene editing therapy targeting telomerase in cancer cells. 

Through the present work, we confirmed that TERT is a valid target to suppress tumor cell survival disruption. Retarded growth and increased cell death were found in the TERT^+/−^ Hela cells. Compared to the WTPE controls, these cells had much lower telomerase activity and much shorter telomeres. These observations are consistent with the canonical pathway of how TERT affects cell growth/survival: TERT→telomerase→telomere lengths→cellular senescence. It should be noted that recent studies have suggested that TERT may affect cell survival, proliferation, chromatin remodeling, and mitochondrial function, among other things, independent of its effects on telomere lengths [38,39]. Therefore, we cannot rule out the possibility that TERT disruption achieved by gene editing may exert its effects on TERT^+/−^ Hela cell survival through telomerase and telomere independent mechanisms. Regardless of its mechanism of action, which requires further studies to fully elucidate, TERT holds as a promising anti-cancer target gene. 

Through the present work, we further demonstrate that TERT haploinsufficiency is a sufficient dose to result in tumor suppression in vitro and in vivo. The complete absence of xenotransplant from TERT^+/−^ Hela cells in nude mice is particularly encouraging, although cautions should be taken as blocking telomerase in cancer cells has been reported to provoke ALT pathways in some cells, which allow those cells to survive and spread in a telomerase-independent manner [40]. A conceptually safer and likely more effective method is to combine telomerase disruption with other anti-cancer drugs. In support of this, the TERT E4 removal strategy, when performed in bulk (i.e., without selection or enrichment of edited cells), did indeed enhance the efficacy of Herceptin, an Food and Drug Administration (FDA) approved monoclonal antibody drug for treating HER2 positive breast cancer [41], in inhibiting the growth of breast cancer SKBR3 cells (Supplementary Figure S3). Follow-up studies are needed to comprehensively assess the efficacy and safety of such combinational strategies. 

The encouraging results of the present work warrant follow up studies in this direction. One feasible strategy readily to be evaluated in preclinical models consists of using adeno-associated virus (AAV) to package CRISPR/Cas9 components that are driven by a tumor specific promoter to target essential tumor survival genes. For delivery, AAV is the dominant cargo used in gene therapy applications nowadays which recently gained the approval from the FDA [42]. To fit the packaging by AAV, saCas9 [43] or other small size gene editing nucleases are to be used. To achieve tumor-specific gene expression, the TERT promoter, pending future modifications to improve its specificity and efficacy, may be a good choice [44]. The Cas9-based cancer therapy may further benefit from its multiplex targeting capacity, which enables simultaneous targeting multiple genes, for example telomerase genes (e.g., TERT), the ALT pathway genes, and other key ones to cancer cell survival pathways. Such multiplex cancer gene disruption strategy, alone or in combination with anti-cancer drugs, is likely to further enhance the efficacy and safety of gene editing therapy towards the cure of cancer. 

Taken together, the present work validated TERT as a good target gene, developed efficient editing strategies on this gene, and determined that TERT haploinsufficiency is a sufficient dosage to suppress cancer cell survival in vitro and in vivo. Availability of these gene editing strategies may facilitate the development of novel therapeutics for treating cancer.

## 4. Materials and Methods

All animal maintenance, care, and use procedures were reviewed and approved by the Institutional Animal Care and Use Committee (IACUC) of the University of Michigan (IACUC#: PRO00007483; approval date: Jan 25 2017). 

### 4.1. Cas9s and Guide RNAs

The spCas9 plasmid DNA (Cat# 42230) [45] was acquired from Addgene (Watertown, MA, USA). Guide RNAs were designed based on the sequence of the targeted locus using an online tool (http://crispor.tefor.net/).

The sequences of gRNAs targeting TERT are shown below. Underlined letters indicate photospacer adjacent motif (PAM) sequences.

sg1: 5′-CGGTGACCGACGCACTGCGGGGG-3′.
sg2: 5′-ACAATCGGCCGCAGCCCGTCAGG-3′.
sg3: 5′-CGCGTACGACACCATCCCCCAGG-3′.
sg4: 5′-TCAGCCAGACAACAGACTAGGGG-3′.
sg5: 5′-CCTCCAGAAAAGCAGCGTGGGGG-3′.

### 4.2. Cells

Hela (Cat# CCL-2) and PANC1 (Cat# CRL-1469) cells were acquired from American Type Culture Collection (ATCC, Manassas, VA, USA) and cultured in Dulbecco’s Modified Eagle Medium (DMEM) (Cat# 11995-065, Thermo Fisher Scientific, Waltham, MA, USA) supplemented with 10% fetal bovine serum (FBS, Cat# SH3008003, Thermo Fisher Scientific). SKBR3 (Cat# HTB-30) cells were acquired from ATCC, and cultured in McCoy’s 5a Medium Modified (ATCC, Cat# 30-2007), supplemented with 10% FBS. SUM159 cells were cultured in Ham’s F12 (Cat#31765035, Thermo Fisher Scientific) supplemented with 5% FBS, 1 µg/mL hydrocortisone (Cat#H0888, Sigma Aldrich, St. Louis, MO, USA), and 5 µg/mL recombinant human insulin (Cat#91077C Sigma Aldrich). Human Aortic Smooth Muscle Cells (HASMC, Cat#CC-2571, Lonza, Alpharetta, GA, USA) were cultured with Clonetics SmGM-2 BulletKit (Cat# CC-3182, Lonza). Human iPSCs were acquired from ATCC (Cat# ACS-1030), and cultured in feeder-free condition in mTeSR1 (85850, StemCell Technologies, Vancouver, BC, Canada) on Matrigel (354277, Corning, Corning, NY, USA)-coated cultureware surface.

### 4.3. Cell Electroporation

All cells except PANC1 and SKBR3 cells were transfected by using a tube electroporation machine (Model#CTX-1500A LE, Celetrix, Manassas, VA, USA). 2–3 × 10^6^ cells were resuspended in 120 µL electroporation buffer (Cat#13–0104, Celetrix) mixed with 6 μg spCas9 expressing pDNAs. The electroporation conditions were 620 V for 30 ms. 

### 4.4. Cell Transfection

PANC1 cells do not tolerate the tube electroporation transfection therefore we used a commercial Lipofectamine 3000 kit (Cat#L3000008, Thermo Fisher Scientific) to transfect about 80% confluence cells (seed 5 × 10^5^ one day before transfection in one well of 6-well plate) with a total of 5 µg spCas9 plasmid DNA, following the manufacturer’s instructions. Lipofectamine transfection was also used on the SKBR3 cells in the experiment to evaluate growth inhibitory activity of Herceptin. 

### 4.5. Determination of Indels by Deep Sequencing

Cells were harvested 48 h after transfection and genomic DNAs were extracted with the Wizard Genomic DNA Purification Kit (Cat#A1120, Promega, Madison, WI, USA). Targeted regions were PCR amplified using high-fidelity PCR master mix (Cat#F532L, Thermo Fisher Scientific) with corresponding primers (Supplementary Table S2). 

The products were gel-purified using Qiaquick gel purification kit (Cat#28706, Qiagen, Germantown, MD, USA). The purified PCR products were sent to the DNA sequencing Core at Massachusetts General Hospital (Cambridge, MA, USA). All deepseq results were analyzed using the NCBI blastn suite tool online (https://blast.ncbi.nlm.nih.gov/Blast.cgi). Indel rate was calculated as the ratio of the cumulative reads over total reads. 

### 4.6. Determination of E4 Deletion

Single cell-derived colonies that were subjected to the dual gRNA Exon removal strategy were used to determine the E4deletion by PCR. The Primers used as shown in Supplementary Table S2. Exon removal efficiency is evaluated with single cell colony genotyping. Briefly, single-cell colonies were established with limited dilution method, and single-cell colonies were randomly picked up and genotyped with PCR. 

### 4.7. Off-Target Analysis

Off-target analysis was carried out similarly as previously described [20]. Briefly, potential off-target loci were predicted by using an online tool (http://crispor.tefor.net). Top nine off-target sites were selected for each gRNA. Each off-target site was amplified with specific PCR primers (Supplementary Table S2), and subjected to T7 endonuclease I (T7EI) assay, in which non-perfectly matched DNA (presumably indel sites) would be recognized and cleaved by T7EI, leading to two cleaved bands; whereas the perfectly matched DNA would not be recognized and cleaved by T7EI hence leading to only one band (the unedited/wild-type band). The ratio of the intensity of the average of the two cleaved bands over that of the wild-type band was used as a quantitative estimate for indel efficiency. 

### 4.8. Western Blot

Western blot analysis of TERT protein levels was conducted by loading 30 μg whole cell lysate onto 4–20% polyacrylamide gels. After being blocked with 5% milk, membranes were incubated with primary antibodies: rabbit anti-TERT (Cat# A2979, Abclonal, Woburn, MA, USA) at 1:1000, or mouse anti-β-actin (Cell Signaling Technology, 3700S) at 1:5000 in 5% milk. The secondary antibodies used were donkey anti-rabbit (LI-COR, 926-32213) at 1:10,000 or donkey anti-Mouse (LI-COR, 926-68072) at 1:10,000 in 5% milk. 

### 4.9. Telomere Content T/S Ratio Assay

T/S ratio assay were carried out by following previous description [27]. Sybr Green quantitative PCR was carried out using primers described in Supplementary Table S2. T/S ratio was calculated by normalization of telomere content with internal control.

### 4.10. Telomerase Activity Assay

The telomerase activity was measured by the Telomerase Repeated Amplification Protocol (TRAP) assay using a commercial kit (TRAPeze Telomerase Detection Kit, Cat#S7700, Millipore, Burlington, MA, USA), following manufacturer’s instructions. 

### 4.11. Cell Death and Apoptosis Assay

A Lactate Dehydrogenase Activity Assay Kit (Cat#MAK066, Millipore) was used to evaluate cell death. An Annexin V FITC staining-based Dead Cell Apoptosis Kit (Cat#V13242, Thermo Fisher Scientific) was used to quantify apoptosis rate. 

### 4.12. Cell Senescence Assay

Cell senescence assay was carried out using the Senescence β-Galactosidase Staining Kit (Cat#9860, Cell Signaling Technology, Danvers, MA, USA) following the manufacturer’s instructions. 

### 4.13. Growth Inhibitory Activity by Herceptin

The breast cancer line SKBR3 cells were plated in a 96-well plate at a concentration of 5 × 10^3^/well. The growth inhibitory effect of trastuzumab (Herceptin, Genentech, San Francisco, CA, USA) was tested at concentrations of 15, 45, 135, 400, 1222, 3666, 11,000 and 33,000 ng/mL, and incubated for 84 h. The medium and trastuzumab were replaced every two days. The cells were counted 4 days post trastuzumab treatment. In the treatment group, the cells were transfected by Lipofectamine 3000 two days before they were used in bulk without any screening, enrichment or sorting for the trastuzumab treatment. 

### 4.14. Xenograft Experiment

Nude mice were purchased from the Jackson Lab (Cat#002019). For each nude mouse, its left hind leg was inoculated with wild-type unedited Hela cells while its right hind leg was inoculated with gene edited TERT^+/−^ Hela cells. For inoculation, approximately 1.0 × 10^6^ cells were resuspended in 200 µL of 1:1 Matrigel (Cat#354230, BD Bioscience, San Jose, CA, USA)/DMEM (Cat#11965-84, Thermo Fisher Scientific) and kept on ice prior to cell injection. These cells with Matrigel were intramuscularly into the hind leg of the animal. Six weeks post inoculation, the animals were humanely euthanized and the xenograft were harvested. 

### 4.15. Statistics

Cell viability and death rates, gene expression levels, and T/S ratio Data are presented as mean ± SEM. Measurements were taken from 3 distinct samples. Unpaired t test (two-tailed) was used to compare data using GraphPad Prism 8 software (GraphPad Software, Inc., San Diego, CA, USA). 

## Supplementary Materials

The following are available online at https://www.mdpi.com/1422-0067/21/2/653/s1, Figure S1. Expressions of telomerase genes and relative telomere contents in different cell lines. Figure S2. Western blot analysis of TERT protein expression. Figure S3. Efficacy of TERT disruption in suppressing growth of breast cancer line SKBR3 cells in vitro. Table S1. Off-target analysis of sg4 and sg5 in Hela cells. Table S2. Primers used in the present work.
