# Supplementary material for: CRISPR/Cas9-Mediated TERT Disruption in Cancer Cells
Source: Int J Mol Sci. 2020 Jan 19;21(2):653. doi: 10.3390/ijms21020653 (PMC7014288; doi:10.3390/ijms21020653)
Supplement: Supplementary file 1 [file ijms-21-00653-s001.pdf]

Supplementary Figures and Tables

Title:

CRISPR/Cas9-Mediated TERT Disruption in Cancer Cells

Authors

Luan Wen [1,#,*], Changzhi Zhao [1,#], Jun Song [1], Linyuan Ma [1], Jinxue Ruan [1], Xiaofeng Xia [2], Y. Eugene Chen [1], Jifeng Zhang [1], Peter X. Ma [3] and Jie Xu [1,*]

Supplementary Figure S1. Expressions of telomerase genes and relative telomere contents in different cell lines. (A) Relative expression levels of TERT and TERC in hiPS, HASMC, Hela, PANC1, and SUM159 cells. (B) Relative telomere content determined by T/S ratio in hiPS, HASMC, Hela, PANC1, and SUM159 cells.

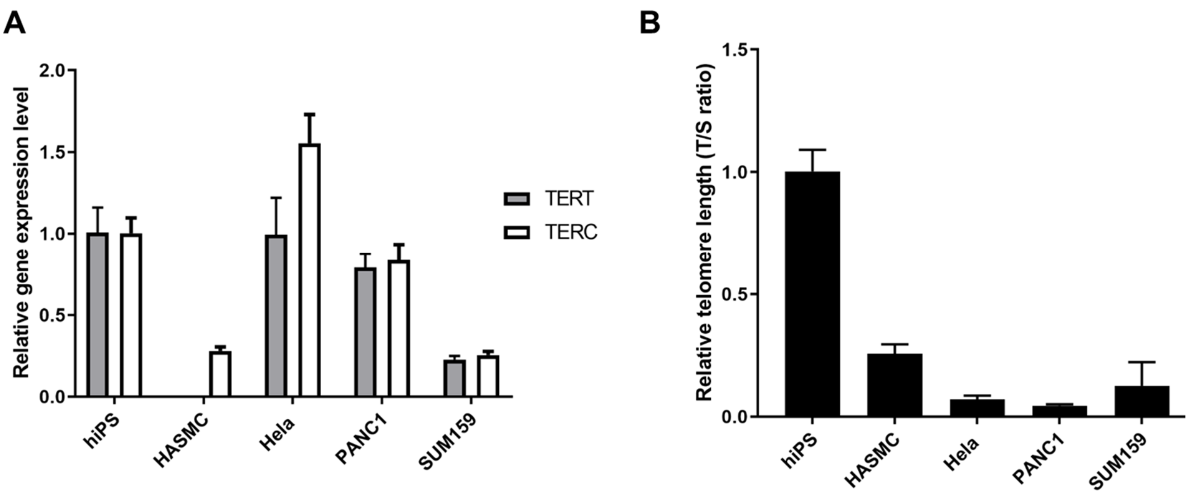

Supplementary S2. Western blot analysis of TERT protein expression in WTPE and Tert$^{+/-}$ Hela and PANC1 cells reveal lower signals in Tert$^{+/-}$ than those in WTPE in Hela (left panel) and PANC1 (right panel) cells.

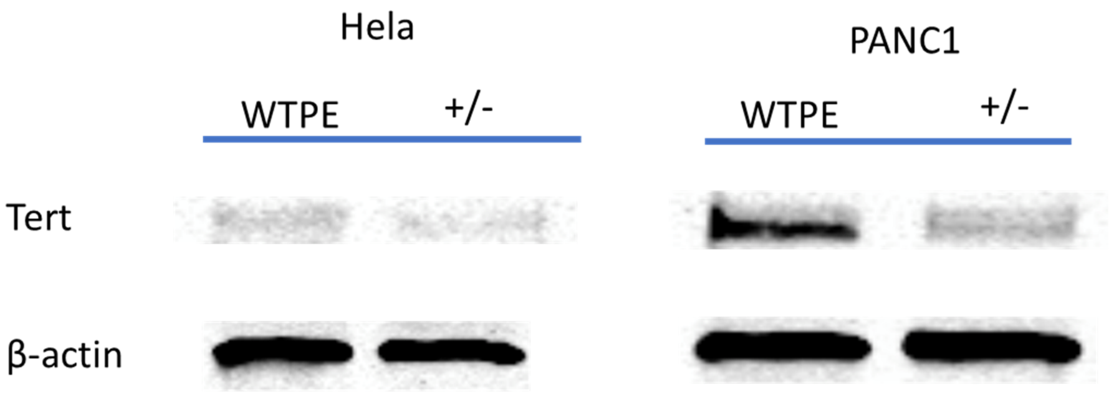

Supplementary Figure S3. Efficacy of TERT disruption in suppressing growth of breast cancer line SKBR3 cells in vitro. (A) Cas9 mediated TERT disruption by sg4 (blue dots) or sg5 (red dots) alone has no additive growth inhibiting effects on SKBR3 cells in the presence of Herceptin (green dots) of concentrations ranging from 0.015 to 33 µg/mL. (B) Cas9 mediated TERT disruption by the exon removal strategy (using both sg4 and sg5, red dots) enhances the efficacy of Herceptin (green dots) of concentrations ranging from 0.4 to 33 µg/mL in inhibiting the growth of breast cancer line SKBR3 cells. * P<0.05. ** P<0.01.

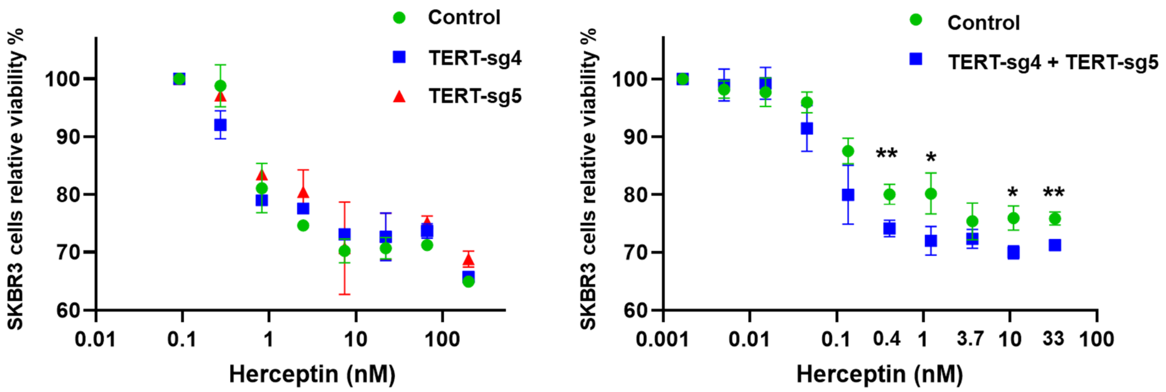

Supplementary Table S1. Off-target analysis of sg4 and sg5 in Hela cells. Blue colored letters indicate the PAM sequence. Red highlighted letters marked the differences of the gRNA with the target sequence. Indel column shows the detected off target results. No off-target edits were detected in all the loci tested for sg4 (n=9) and sg5 (n=9).

**Genomic location**	**Locus details**	**Sequence**	**Indel**
**sg4**			
on target	Intron: Tert	TCAGCCAGACAACAGACTAG GGG	
off-targets			
Chr9: 110141708-110141730	intron: PALM2-AKAP2/AKAP2	TGAGTCACACAACAAACTAG TGG	no
Chr4: 162701165-162701187	intergenic: FSTL5-AC005150.3	TCAGCAAGACGGCTGACTAG AGG	no
Chr5: 171194393-171194415	intron: RANBP17	ACAGACAGAAAACAGATTAG TGG	no
Chr4: 113606577-113606599	intron: CAMK2D	ACAGAAAGACAACAGACTAC GGG	no
Chr2: 235792702-235792724	intergenic: AC064874.1-AGAP1	TGAGCTAGAAAACAGACGAG AGG	no
Chr11: 102361958-102361980	intron: BIRC2	TCAGCAAGACAGCAGACTAG GAG	no
Chr1: 89114677-89114699	intron: GBP2	TAAGCCAGACACCAGACTAG TGA	no
Chr14: 74532057-74532079	intron: LTBP2	TCAGCCAGACATCAGAGCAG AGG	no
Chr7: 96705111-96705133	intron: SHFM1	TGAGGCAGACAACAGACTGG TGA	no
**sg5**			
on target	Intron: Tert	CCTCCAGAAAAGCAGCGTGG GGG	no
off-targets			
Chr5: 61144587-61144609	intergenic: CTC-436P18.4-NDUFAF2	GCTCCAGAGAAGCAGAATGG TGG	no
Chr11: 127252553-127252575	intergenic: CTD-2234N14.1-RP11-480C22	CCTCCTGGAAAGCAAAGTGG GGG	no
Chr4: 156257157-156257179	intergenic: CTSO-AC108211.1	GCTCCAGAAGAGCAGCGTGG TGG	no
Chr10: 104560185-104560207	intergenic: RP11-127O4.3-RP11-127O4.2	CCTCCAGCATAGCAGCGTGG GGG	no
ChrX: 4912396-4912418	intergenic: AC074035.1-SNORA20	TCTCCAGAACAGCAGCATGG GGG	no
Chr11: 16481443-16481465	intergenic: SOX6-RN7SL188P	CTTCCAGAACAGCAGAGTGG TGG	no
Chr17: 18017527-18017549	intergenic: LRRC48-ATPAF2	CCTCCAGAAGAGCAGAGTGC AGG	no
Chr8: 89901981-89902003	exon: OSGIN2	CTTCCATAAAAGAAGCGTGG TGG	no
Chr17: 3515883-3515905	intron: TRPV3	CCTCCAAAAAAGCAGAGAGA GGG	no

Supplementary Table S2. Primers used in the present work.

**Primers for amplification of gRNA edited sequences**	
Primers for gRNA1 edited products:	Forward 5′-AAGAACGTGCTGGCCTTCG-3′<br>Reverse 5′-AGCCACCAGCACAAAGAGC-3′
Primers for gRNA2 edited products	Forward 5′-GGGGCTCAAACGCACTTCT-3′<br>Reverse 5′-ACGTCCAGACTCCGCTTCAT-3′
Primers for gRNA3 edited products	Forward 5′-CGTGAACCTTACGTGGCTCTT-3′<br>Reverse 5′-ACTCACACAGGTGGATGTGAC-3′
Primers for gRNA4 edited products	Forward 5′-AGCTGATTCTGCTCCTTGGC-3′<br>Reverse 5′-TCCCACGACGTAGTCCATGT-3′
Primers for gRNA5 edited products	Forward 5′-GTCTGTTGTCTGGCTGAGCA-3′<br>Reverse 5′-GTTTGAAACGGGTTCCTGGC-3′
**Primers for detection of E4 removal**	
	Forward 5′-GAAGGGATGTGGCTCTTTCTACCTG-3′<br>Reverse 5′-CTCACCCCACACTCTCCTCAGATG-3′
**Primers for T/S ratio analysis**	
Primers for T/S ratio	Forward 5′-GGTTTTTGAGGGTGAGGGTGAGGGTGAGGGTGAGGGT-3′<br>Reverse 5′-TCCCGACTATCCCTATCCCTATCCCTATCCCTATCCCTA-3′
Primers for internal control 36B4	Forward 5′-CAGCAAGTGGGAAGGTGTAATCC-3′<br>Reverse 5′-CCCATTCTATCATCAACGGGTACAA-3′
**Primers for off-target (OT) analysis**	
Sg4-OT1	Forward 5′-TGTTGTCCATCAGGCTTCCC-3′<br>Reverse 5′-CATTTCCACACCAGGGGGAT-3′
Sg4-OT2	Forward 5′-GATAGCTTGGGGCACTGTGA-3′<br>Reverse 5′-GATAGCTTGGGGCACTGTGA-3′
Sg4-OT3	Forward 5′-AGCTAATCACCTCCGCTGTG-3′<br>Reverse 5′-TTCCCCCAACTCCCCAGTAA-3′
Sg4-OT4	Forward 5′-TCCCTGCGTTCCAATAACCTC-3′<br>Reverse 5′- TTGCTCATGTGGCCGTAGTT-3′
Sg4-OT5	Forward 5′-CTGTGGTGTGTTAGGGGCAT-3′<br>Reverse 5′-CATGGTCACTGGGAGTGGTC-3′
Sg4-OT6	Forward 5′-AAGCTGTCAGTGTCCTCTGC-3′<br>Reverse 5′-AGCGTCTGCACCTACAAAGG-3′
Sg4-OT7	Forward 5′-CTGTCTCCAGCGTTTCGAGT-3′

	Reverse 5′-CACTGCTGATGGCATTGACG-3′
Sg4-OT8	Forward 5′-GGTGGACAGTGGGAGACAAG-3′<br>Reverse 5′-CCCTGGAAACCACAGAGGTC-3′
Sg4-OT9	Forward 5′-GCAAAAGCCCTTTTTGGCAC-3′<br>Reverse 5′-CATACGGTCAGGTTGGTGGT-3′
Sg5-OT1	Forward 5′-TGGGCCTAAGACTGACTCCT-3′<br>Reverse 5′-CTGGGCTTTGGAAATGCACTC-3′
Sg5-OT2	Forward 5′-AGAGGTTGGAGATGCCTTGG-3′<br>Reverse 5′-CTGAACTAGGCCAGGAGGTTG-3′
Sg5-OT3	Forward 5′-ATATGCACCTGCATGCCCTG-3′<br>Reverse 5′-GGACCACCCTGTCACTCCTA-3′
Sg5-OT4	Forward 5′-GCCACTTGCTCTACGTCACA-3′<br>Reverse 5′-GGAGTCCATCCTCTGGTCCT-3′
Sg5-OT5	Forward 5′-GCTGGTCTAGGCTTTGCTTG-3′<br>Reverse 5′-GGGAGGCAGAAAAGAGTGTG-3′
Sg5-OT6	Forward 5′-TCAAGTGGGTTTTGCAGGAGA-3′<br>Reverse 5′-CCAAGTTCATTCCAACCACCATC-3′
Sg5-OT7	Forward 5′-TGGCCAGCAGTGAATGTAGG-3′<br>Reverse 5′-TTGAGATGAACAGCCGGTCC-3′
Sg5-OT8	Forward 5′-TGCATAACTCTGGGGCAGTC-3′<br>Reverse 5′-CGGCAATCGAGACCTAGACC-3′
Sg5-OT9	Forward 5′-GATTCCGGATGGGAGAGCTG-3′<br>Reverse 5′-GGTAGGAGGAGGTGGGCTTA-3′
